# Severe necrotizing tracheobronchitis caused by influenza B and methicillin-resistant *Staphylococcus aureus* co-infection in an immunocompetent patient

**DOI:** 10.1186/s12941-024-00715-1

**Published:** 2024-06-17

**Authors:** Shu Wang, Jianhua Yang, Wenwu Sun, Yang Tao

**Affiliations:** 1 Department of Intensive Care Medicine, Chongqing University Central Hospital, Chongqing Emergency Medical Center, Chongqing, 400016 China; 2 Departments of Emergency, Ruijin Hospital, School of Medicine, Shanghai Jiao Tong University, Shanghai, 200025 China

**Keywords:** Necrotizing tracheobronchitis, Influenza B, *Staphylococcus aureus*, Next-generation sequencing, Vancomycin

## Abstract

**Purpose and method:**

Necrotizing tracheobronchitis is a rare clinical entity presented as a necrotic inflammation involving the mainstem trachea and distal bronchi. We reported a case of severe necrotizing tracheobronchitis caused by influenza B and methicillin-resistant *Staphylococcus aureus* (MRSA) co-infection in an immunocompetent patient.

**Case presentation:**

We described a 36-year-old man with initial symptoms of cough, rigors, muscle soreness and fever. His status rapidly deteriorated two days later and he was intubated. Bronchoscopy demonstrated severe necrotizing tracheobronchitis, and CT imaging demonstrated multiple patchy and cavitation formation in both lungs. Next-generation sequencing (NGS) and bronchoalveolar lavage fluid (BALF) culture supported the co-infection of influenza B and MRSA. We also found T lymphocyte and NK lymphocyte functions were extremely suppressed during illness exacerbation. The patient was treated with antivirals and antibiotics including vancomycin. Subsequent bronchoscopy and CT scans revealed significant improvement of the airway and pulmonary lesions, and the lymphocyte functions were restored. Finally, this patient was discharged successfully.

**Conclusion:**

Necrotizing tracheobronchitis should be suspected in patients with rapid deterioration after influenza B infection. The timely diagnosis of co-infection and accurate antibiotics are important to effective treatment.

## Introduction

Necrotizing tracheobronchitis is a rare clinical entity presented as a necrotic inflammation involving the mainstem trachea and distal bronchi. Most necrotizing tracheobronchitis is usually developed in patients with immunocompromised conditions, like long term mechanical ventilation [1], low birth weight infant [2], metastatic carcinoma or use of immunosuppressants [3]. However, especially in immunocompetent patients, necrotizing tracheobronchitis associated with influenza is very rare. Most of the published cases reported the necrotizing tracheobronchitis with influenza A only [4], or the complication of influenza A with bacterial co-infection [5–7]. There are few reported instances of co-infection involving other types of influenza and bacteria in necrotizing tracheobronchitis. Here we report a case of necrotizing tracheobronchitis caused by influenza B and methicillin-resistant *Staphylococcus aureus* (MRSA) co-infection in an immunocompetent patient.

## Case report

**Fig. 1:**
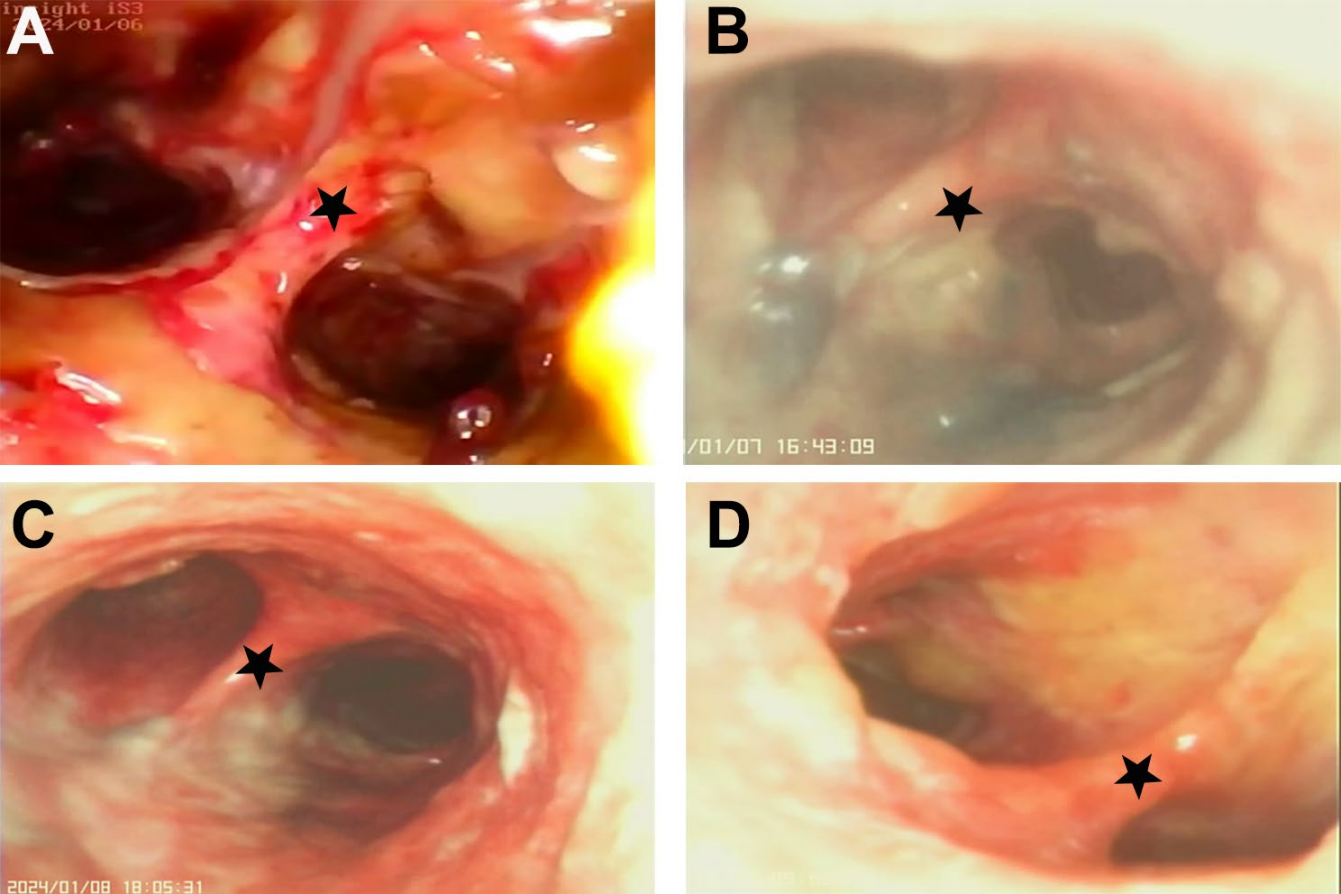
Follow-up bronchoscopy. (**A**) Severe hemorrhagic tracheobronchitis involving the mainstem trachea and distal bronchi with sloughing and diffuse mucosal swelling observed on the 2nd day after admission. (**B**) Severe mucosal inflammation and purulent secretion were observed on the 3rd day after admission. (**C**) Inflammatory mucosal change with reduced secretion on the 4th day after admission, one day after switching to vancomycin. (**D**) The affected mucosa resolved on the 7th day after admission, four days after switching to vancomycin. Carina (black star)

**Fig. 2:**
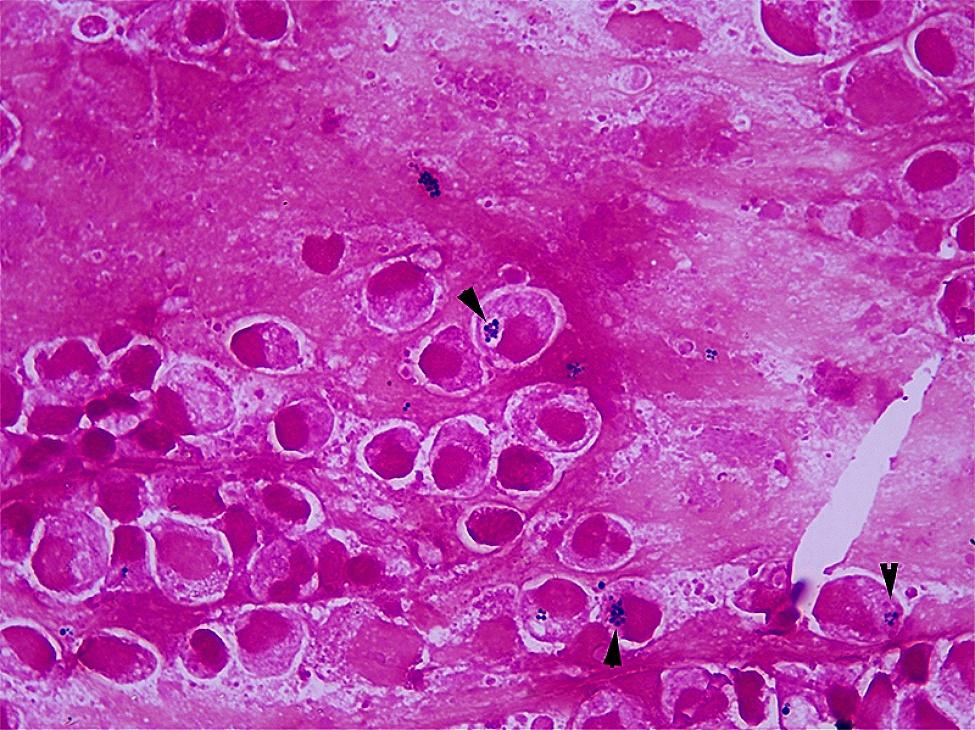
Bronchoalveolar lavage fluid Gram stain showed Gram positive cocci phagocytosed by white blood cell (black arrow)

**Fig. 3:**
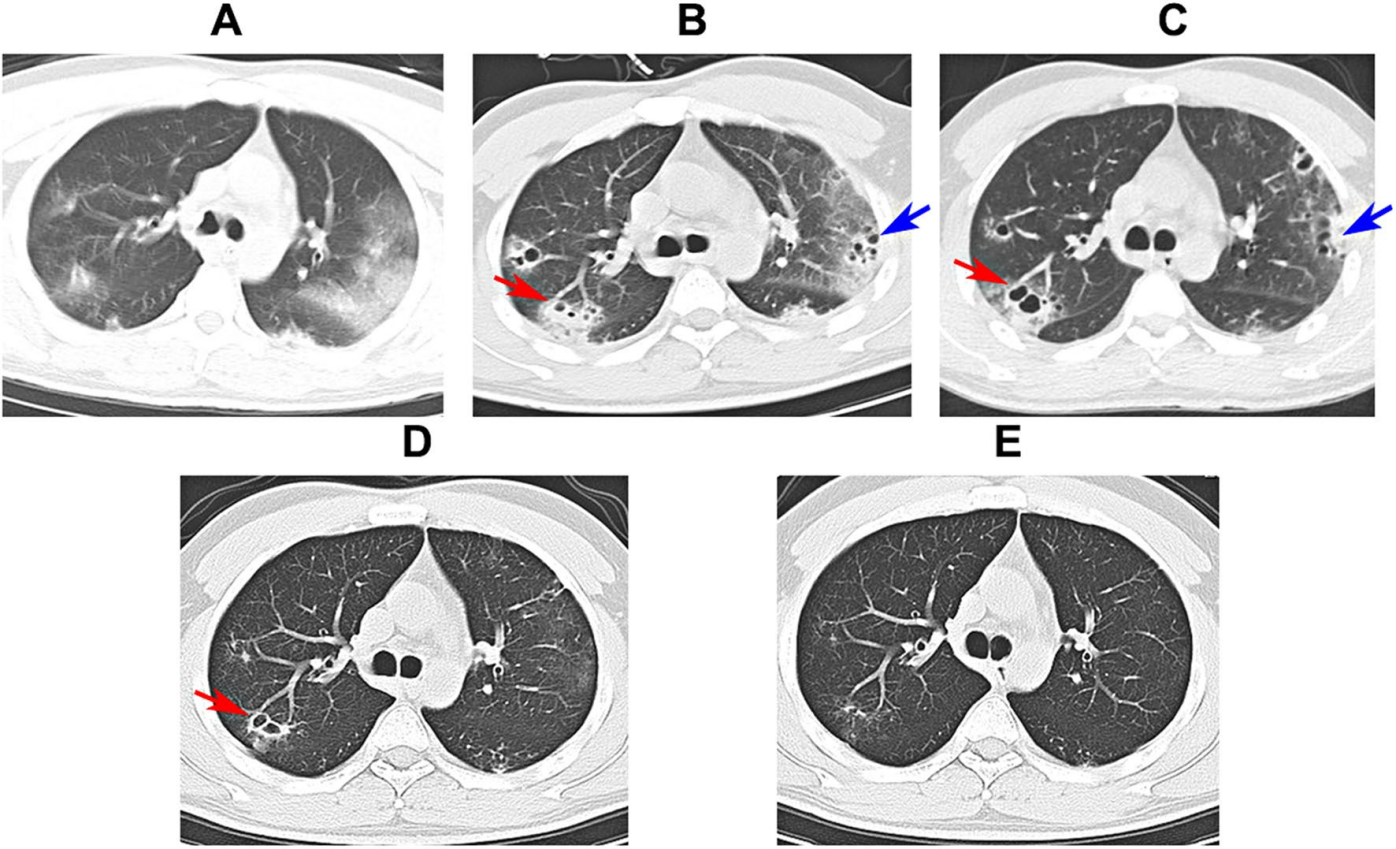
Chest computed tomography images. (**A**) Patchy consolidations in both lungs observed on the 1st day after admission. (**B**) Multifocal nodular opacities with new cavitations in left lung (blue arrow) and right lung (red arrow) observed on the 8th day after admission. (**C**) Residual cavitations in left lung (blue arrow) and enlarged cavitations in right lung (red arrow) observed on the 15th day after admission (discharge). (**D**) Resolved cavitations in left lung and resolved patchy consolidations with residual cavitations in right lung (red arrow) observed on the 4th day after discharge. (**E**) Resolved cavitations in both lungs observed 1 month later after discharge

**Fig. 4:**
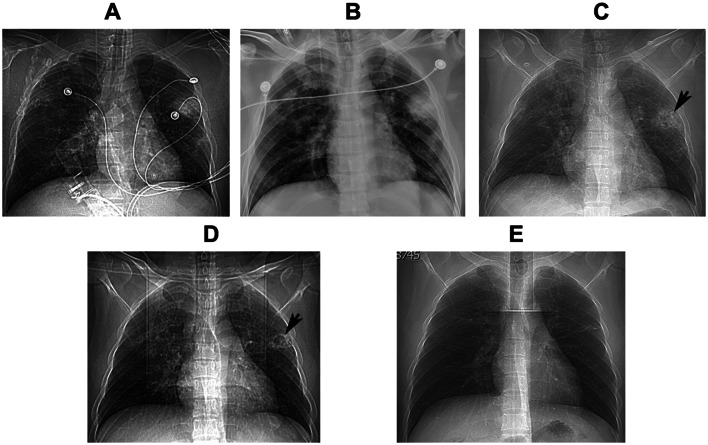
Plain chest radiographs. (**A**) On the 1st day after admission. (**B**) On the 4th day after admission. (**C**) Multiple small cavitations in nodular opacities in right upper lung (black arrow) observed on the 8th day after admission. (**D**) A large cavitation formed in right upper lung (black arrow) observed on the 15th day after admission (discharge). (**E**) one month later after discharge

A 36-year-old man with no underlying history or tobacco use presented to the emergency department of local hospital with 3-days history of cough, rigors, muscle soreness and fever. Vital signs: blood pressure (BP) 133/75 mmHg, pulse rate (PR) 105 beats per minute, respiratory rate (RR) 19 breaths per minute, temperature (T) 39.0 °C, and SpO_2_ 97% on air. Rapid influenza test using colloidal gold assay was positive for influenza B. Routine blood test showed normal white blood cell (WBC) count with neutrophils 74.6%, but decreased lymphocyte of 0.63 × 10^9^/L (normal range 1.10 × 10^9^/L − 3.20 × 10^9^/L). Empiric ibuprofen and oseltamivir were started. However, the symptoms aggravated with dyspnea and chest pain. The patient presented to the emergency department 2 days later. Vital signs: BP 122/86 mmHg, PR 132 beats per minute, RR 37 breaths per minute, T 37.5 °C, and SpO_2_ 78% on air. Coarse breathing sounds were noted in both lung fields on auscultation. He was admitted, and polymerase chain reaction (PCR) test of the throat swabs confirmed the influenza B infection. Laboratory tests showed: WBC 5.87 × 10^9^/L, neutrophils 80.7%, platelet 118 × 10^9^/L, lymphocyte 0.41 × 10^9^/L, creatinine 88 umol/L, blood urea nitrogen 4.96 mmol/L, alanine aminotransferase 25 IU/L, total bilirubin 37.8 umol/L, direct bilirubin 7.8 umol/L, procalcitonin 5.33 ng/mL, lactate 1.9 mmol/L, respectively. The proBNP and cTNI levels were normal. Arterial blood gas showed pH 7.52, PCO_2_ 33 mm Hg, PO_2_ 60 mm Hg, and bicarbonate 28.1 mmol/L on mask of 10 L/min oxygen. Invasive mechanical ventilation was needed 4 h after admission due to acute respiratory distress syndrome (ARDS), and was started on imipenem 2.0 g q6h, moxifloxacin 0.4 g qd, Baloxavir marboxil 40 mg qd, methylprednisolone, and prone position ventilation. Bronchoscopy showed severe hemorrhagic tracheobronchitis involving the mainstem trachea and distal bronchi with sloughing and diffuse mucosal swelling (Fig. 1A). Bronchoalveolar lavage fluid (BALF) was sent for next-generation sequencing (NGS) and culture. *Staphylococcus aureus* (homogenized sequence 11,597, 3,134,324 copies/mL) and influenza B (homogenized sequence 25, 6757 copies/mL) were detected from NGS, and other bacteria, fungi, and parasites were not detected. Methicillin-resistant *Staphylococcus aureus* (MRSA), which was susceptible to vancomycin, linezolid, and teicoplanin by drug sensitivity test, was isolated from the culture. BALF Gram stain showed Gram positive cocci phagocytosed by white blood cells (Fig. 2). Chest computed tomography (CT) and plain chest radiographs demonstrated patchy consolidations and multifocal nodular opacities with cavitations in both lungs (Fig. 3 and Fig. 4). Consecutive multiple sputum specimens yielded the same cultures of MRSA. The imipenem was de-escalated to piperacillin/tazobactam 4.5 g q8h, and moxifloxacin was switched to vancomycin 1.0 g q12h after the NGS reports on the 3rd day after admission, with the maintenance of other treatment plans. Bronchoscopy showed much improvement during follow-up (Fig. 1B-D). The methylprednisolone was stopped on the 4th day after admission; the patient was extubated on the 7th day after admission; Baloxavir marboxil and piperacillin/tazobactam was stopped on the 8th day after admission; Vancomycin was switched to oral linezolid on the 9th day after admission. Finally, he was discharged to home on the 15th day after admission. The patient’s lymphocyte count was followed until discharge, and lymphocyte subsets were measured using the FACSCanto Plus flow cytometer (BD Biosciences). In lymphocyte subsets, CD3 + CD8 + T cell, CD3 + CD4 + T cell, and CD3-CD16+/56 + NK cell counts are extremely low on the 3rd day after admission (Table 1). After antibiotics adjustment focusing on MRSA, these indices gradually recovered and restored to normal range on the 12th day after admission.

**Table 1:** lymphocyte subsets from the day after admission

		3rd	8th	12th	15th
Item	Normal range				
CD3 + T cell count	834–2217 count/uL	236	451	1085	1019
CD3 + CD8 + T cell count	269–1059 count/uL	87	174	394	403
CD3 + CD4 + T cell count	395–1265 count/uL	81	204	483	405
CD3-CD16+/56 + NK cell count	136–880 count/uL	1	32	141	153
CD3-CD19 + B cell count	92–498 count/uL	132	184	263	163

Lymphocyte subsets were measured using the FACSCanto Plus flow cytometer (BD Biosciences)

## Discussion

Influenza virus infection is mainly prevalent in the winter season. Mostly, the symptoms are self-limiting and mild, but influenza can also lead to life threatening pneumonia and ARDS. There are several influenza A pandemics in history [8]. However, the awareness of influenza B infection is less than influenza A due to its lack of pandemic potential. Infections caused by Influenza B were previously believed to lead to a milder severity than influenza A, but the published data suggest that the rate of mortality may be similar between these two influenza types in the overall population [9].

Necrotizing tracheobronchitis is a rare complication usually presumed to be caused by a non-infectious origin, or a secondary bacterial infection following a primary viral respiratory infection. It has been reported in autopsy studies involving pandemic 1918 H1N1 and pandemic 2009 H1N1 influenza patients [10, 11]. Co-infection with bacteria commonly occurs in critically ill patients infected with influenza. Necrotizing tracheobronchitis due to co-infection of the influenza A virus and MRSA has been reported in several cases [5–7, 12]. Unlike these previous cases, there are few reported instances of co-infection involving other types of influenza and bacteria. Only one case reported co-infection of the influenza B virus and MRSA in an old man with a history of chronic diseases [13]. Complication rates in influenza infection are highest in patients with underlying chronic diseases, immunosuppressed status, or older subjects. However, no predisposing factors could be identified. Interestingly, in all the previously reported necrotizing tracheobronchitis cases following the influenza infection in immunocompetent patients, including in this case, *Staphylococcus aureus* is the most commonly detected co-infection bacteria. It has been well known that culturing positive rate of Staphylococcus aureus is high in critically ill patients infected with influenza, and is associated with an increased risk of death [14]. It is still unexplained why the complication rate of necrotizing tracheobronchitis is high in influenza and *Staphylococcus aureus* co-infection. In previous reports, the reasons may be the toxic shock syndrome toxin-1 (TSST-1) or panton-valentine leukocidin (PVL) expressed by some hypervirulent *Staphylococcus aureus* strains [7, 15]. Lymphocyte function is very important in host defense against pathogenic microorganism. In this case, the lymphocyte subsets may indicate the attenuated T lymphocyte and NK lymphocyte functions by the synergy of influenza and *Staphylococcus aureus*, which is supported by published studies [16, 17].

In this case, early empirical antiviral treatment and antibiotic treatment with anti-Gram negative antibiotic combined with anti-Gram positive antibiotic was administered when this patient was suspected of co-infection. Empirical using of vancomycin or linezolid should be considered in patients suspected of Gram positive cocci infection. It is recommended that antibiotics regimen should be changed according to the culture results of high-quality respiratory specimens. In this case, intravenous vancomycin has a significant clinical effect in the treatment of MRSA tracheobronchitis.

In conclusion, here we report a young immunocompetent patient with severe necrotizing tracheobronchitis caused by influenza B and methicillin-resistant *Staphylococcus aureus* co-infection. Particularly, we find T lymphocyte and NK lymphocyte functions are extremely suppressed during illness exacerbation. Necrotizing tracheobronchitis should be suspected in patients with rapid deterioration after influenza B infection. The timely diagnosis of co-infection and accurate antibiotics are important to effective treatment.

## Data Availability

All relevant data has been presented in the manuscript and further inquiry can be directed to the corresponding author.
